# Feasibility of an Upper Limb Strength Training Program in Persons with Spinal Cord Injury during Primary Rehabilitation—An Uncontrolled Interventional Study

**DOI:** 10.3390/ijerph192214743

**Published:** 2022-11-09

**Authors:** Claudio Perret, Jolien De Jaegher, Inge-Marie Velstra

**Affiliations:** 1 Sports Medicine, Swiss Paraplegic Centre, 6207 Nottwil, Switzerland; 2 Ambulatory Physiotherapy, Swiss Paraplegic Centre, 6207 Nottwil, Switzerland; 3 Clinical Trial Unit, Swiss Paraplegic Centre, 6207 Nottwil, Switzerland

**Keywords:** exercise, training, paraplegia, physical therapy, performance

## Abstract

Data concerning the outcomes of standardized strength-training programs in people with acute spinal cord injury (SCI) are scarce. The present study evaluated the feasibility and effects of a clinic-internal strength-training concept in people with paraplegia during the course of primary rehabilitation. For this purpose, participants followed a 10–12 week standardized supervised strength-training program (30 training sessions) during primary rehabilitation. At the beginning, 5–6 weeks and 10–12 weeks later, maximal strength based on indirect one-repetition maximum (1RM) measurements for two specific exercises (triceps press; horizontal rowing pull) was determined. Twelve out of 17 participants successfully completed the study. Maximal weights for 1RM significantly increased over the 10–12 week training program for the triceps press (+30%; *p* = 0.018) and the horizontal rowing pull (+41%; *p* = 0.008). Training compliance was 95%. Reasons for study exclusion were urgent surgery (*n* = 2), cardio-respiratory complications (*n* = 1), shoulder pain (*n* = 1) and a training compliance of less than 50% (*n* = 1). In conclusion, a supervised and standardized strength-training program during primary rehabilitation of people with paraplegia is feasible and leads to significant increases in maximal strength. Although study participants showed a high training compliance, factors such as medical complications may impede the proper implementation of a strength-training concept into daily clinical practice.

## 1. Introduction

One of the ultimate goals of the rehabilitation process of people with spinal cord injuries (SCIs) is to regain the highest possible functioning, mobility and independence [1]. A higher fitness level seems to be significantly related to a better quality of well-being in individuals with SCI [2] and supports the prevention of common secondary complications such as cardiovascular diseases, diabetes [3,4], obesity [5] and depression [6]. In fact, several studies have reported that strength and endurance training on a regular basis lead to significant improvements in strength and physical fitness in individuals with SCI [7,8,9,10]. The consequence of these improvements is a positive impact on the functioning and participation of these individuals, leading to a better quality of life [8,11,12,13,14,15,16,17]. In addition to these positive effects, a recently published review article [18] concluded that strength training in individuals with SCI is safe and feasible.

With regard to strength training in people with SCI, several training recommendations for this population exist. A common suggestion is two- to three-weekly training sessions including three sets of 8 to 12 repetitions per set with weights around 50–80% of the individual’s one repetition maximum (1RM). During such a training session, the big muscle groups—which are important for activities of daily life—should be preferentially trained [5,9,19,20,21,22,23]. However, several reviews [9,18,24] have emphasized that these training recommendations are mainly based on studies with individuals suffering from chronic SCI. Thus, there seems to be an urgent need to also investigate such training recommendations and their effects in individuals with acute SCI and during early rehabilitation [9], as these matters have not been clearly established during the early stages of SCI by the existing literature so far.

Based on the above-mentioned recommendations, a general clinic-internal 10- to 12-week strength-training concept for our patients with acute SCI in the primary rehabilitation process was developed some years ago. Thereby, the goal was to start the training program as soon as possible after admission to the clinic and to consciously apply it to the majority of our patients, independent of the completeness or level of the lesion. Although this concept was already implemented some years ago, its particular training effects and feasibility have not been elucidated so far. Therefore, the aim of the present study was to observe the feasibility of our clinic-internal strength-training concept by investigating the development of maximal strength in people with SCI during the early rehabilitation process. Furthermore, factors which delay or impede a consequent strength gain based on a structured training concept were to be identified.

## 2. Materials and Methods

### 2.1. Study Participants

**Figure 1:**
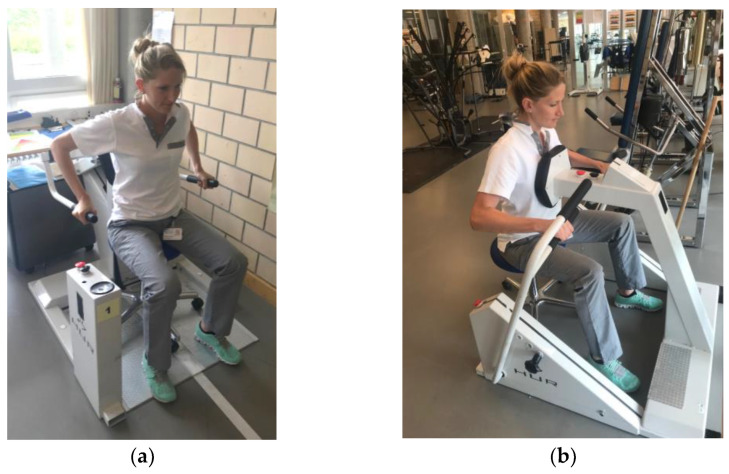
Triceps press (**a**) and horizontal rowing pull (**b**) machine used for training.

Participants undertaking primary rehabilitation at our SCI-specialized clinic were recruited from October 2018 to February 2019. Participants had to suffer from a traumatic or non-traumatic paraplegia and be able to perform strength-training exercises on a triceps press and a horizontal rowing pull machine (Figure 1). The minimal age for inclusion was 18 years. Exclusion criteria were polyneuropathies, degenerative or progressive illnesses and diagnosed cognitive limitations. A detailed description of the study participants can be found in Table 1. The study was conducted in accordance with the Declaration of Helsinki, and approved by the responsible Ethics Committee of Ethikkommission Nordwest- und Zentralschweiz, Basel, Switzerland (Approval-Nr.: 2018-01562). Additionally, participants gave their written informed consent before study participation.

### 2.2. Study Design and Recruitment

**Figure 2:**
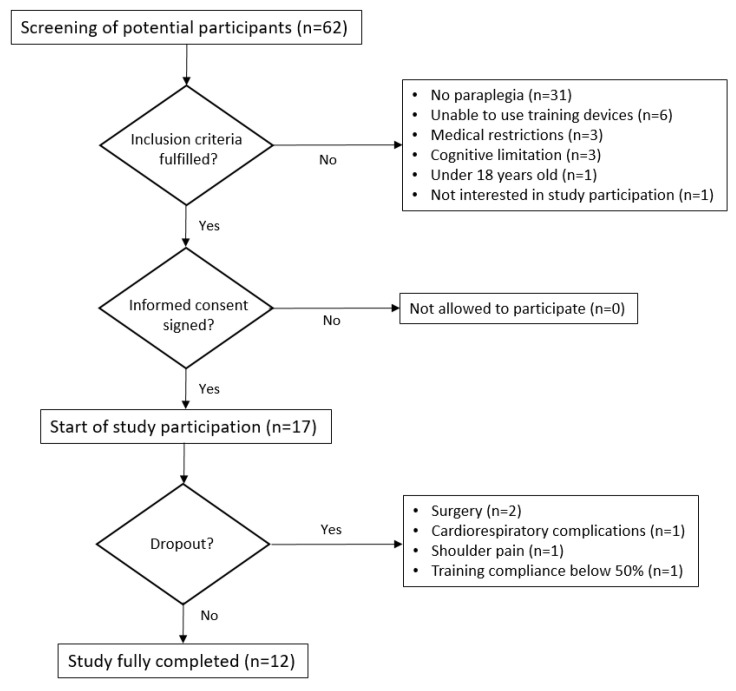
Flow chart of the recruitment process.

A prospective longitudinal monocentric study was performed. For this purpose, patients suffering from SCI who were undergoing primary rehabilitation were screened by an authorized study physiotherapist for in- and exclusion criteria. As one goal of our concept was to consciously apply it to the majority of our patients, independent of the severity or level of the lesion, each potential study participant was screened and included if written informed consent was obtained. A detailed flow chart of the recruitment process can be found below (Figure 2).

### 2.3. Procedures

A trained physiotherapist performed all measurements of the one repetition maximum (1RM) according to a standardized procedure and supervised the strength-training process over the whole duration of the study (for more details see below).

### 2.4. Strength Training and Determination of the 1RM

The strength training of all participants included performing exercises on a triceps press (Dip/Shrug Easy Access, Ab Hur Oy, Kokkola, Finland) as well as on a horizontal rowing pull machine (Lat Pull Easy Access, Ab Hur Oy, Kokkola, Finland) over a period of 10–12 weeks. The maximum force was determined at the start of the study (T1) and the follow-up timepoints after 5–6 (T2) and 10–12 weeks (T3) for both the triceps press and the horizontal rowing pull machine measurements performed and expressed via the indirect 1RM. For this purpose, participants started with a specific warm-up, performing 15 repetitions with a very low weight. Thereafter, the weight was increased to a level at which a maximum of 20 repetitions was possible. The weight had to be moved over the whole range of motion at a given rhythm (one second per movement direction). This procedure was done for both devices used, with a break of 10 min between exercises. The 1RM was calculated indirectly via the maximum number of repetitions achieved at the given load. For this purpose, the validated prediction formula of Brzycki (1RM = number of repetitions/(1.0278 − (weight[kg] × 0.0278)) was used in this study [25]. The calculated 1RM was collected metrically in kilograms (kg). In order to capture the development of the maximum force, the differences in the 1RM between the measurement time points were also calculated in absolute values (kg). Measurements to determine the 1RM were always performed at the same time of day and in the same order.

**Figure 3:**
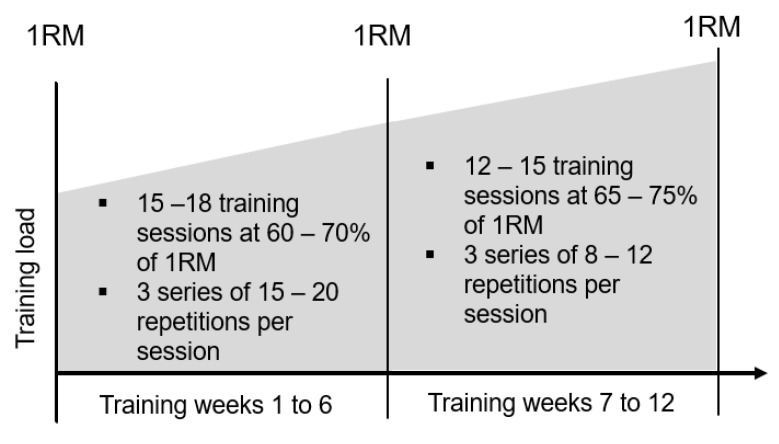
Study design.

Based on data obtained at the time point T1, participants trained for the following 5–6 weeks at approximately 60–70% of their corresponding 1RM three to four times a week (15–18 training sessions in total). For each exercise, three sets with a break of about two minutes between sets were completed. The goal during this training phase was to reach complete muscle fatigue at the end of a set within 15–20 repetitions. In case 20 repetitions were reached, the weight was increased by about 3–5% for the next training session. Between the time points T2 and T3, the strength-training frequency was reduced to two to three sessions a week (for 12–15 sessions in total). The goal during this time period was to reach total muscle fatigue at the end of a set within 8–12 repetitions. In case 12 repetitions were reached, the weight was increased by about 3–5% for the following training session. Figure 3 shows an overview of the whole training process. A training compliance of 100% corresponded to 30 strength-training sessions within 10–12 weeks. Weights were periodically (about every two weeks) adjusted by the physiotherapist. In case of a missed training session, the reason for the absence was documented.

### 2.5. Data Analysis

A commercially available software package (SPSS Statistics for Windows, IBM Corporation, New York, NY, USA) was used for data analysis. Demographic and medical data on AIS grades and lesion levels were presented descriptively. Only complete data sets were analyzed. Causes of training failure were also reported. Training failure was defined as a training gap of more than 14 consecutive days.

According to a Kolmogorov–Smirnov test, the data were not normally distributed and therefore were presented as median (minimum; maximum). A Friedman test was used to detect differences for the 1RMs of the two testing conditions (triceps press and horizontal rowing pull), comparing the three time points T1 to T3. The significance level was set at α < 0.05. In case of a significant difference, a Wilcoxon post-hoc analysis with Bonferroni correction was applied. The effect sizes for the Friedman tests were calculated by Kendall’s W followed by Cohen’s d. The level of agreement for the effect sizes was assessed according to their classification by Cohen, with 0.1–<0.3 being a “small effect”, 0.3–<0.5 being a “moderate effect” and ≥0.5 being a “large effect”.

## 3. Results

### 3.1. Study Participants

In total, 12 of the original 17 participants enrolled at the start of the study, successfully completed all 1RM measurements and were included for data analysis. The dropout reasons of the remaining five study participants were surgeries (n = 2), cardiorespiratory complications (n = 1), shoulder pain (n = 1) and a training compliance below 50% (n = 1). During the recruitment period, an additional 42 patients entered our clinic for primary rehabilitation who did not meet the inclusion criteria for this observational study. The 12 participants who completed the study showed a high training compliance of 95%. The median number of days for the baseline measurement (T1) was 38 days (minimum 11, maximum 104) post-SCI. Thereafter, the 10–12 week strength-training program started. The medians for the follow-up measurements of the 1RM during the course of the strength-training program were 43 (31;61) days and 83 (57;98) days after T1 for T2 and T3, respectively.

### 3.2. Changes in Maximal Strength

Significant gains in maximal strength (1RM) were found for the triceps press and the horizontal rowing pull (Table 2) over the 12-week intervention period. Comparing changes between the different measurement time points, significant differences were also present for the rowing pull between T1 and T2 (*p* = 0.012), and for both devices between T2 and T3 (triceps press: *p* = 0.024; horizontal rowing pull: *p* = 0.009), as well as between T1 and T3 (triceps press: *p* = 0.018; horizontal rowing pull: *p* = 0.008). The median 1RM gain between T1 and T3 was 30% (19.8 kg) for the triceps press and 41% (15.9 kg) for the rowing pull.

## 4. Discussion

The present study investigated changes in maximal strength during a 10–12 week standardized clinic-internal training program during primary rehabilitation in people with acute paraplegia. Significant median improvements of 30% and 41% for the triceps press and the horizontal rowing pull were found (Table 2), which are—according to the calculated effect sizes—not only meaningful from a statistical but also from a clinical point of view. Similar increases in maximal strength were found in other studies in which a circuit training regime (combination of strength and endurance exercises) was applied. Jacobs and colleagues [26] reported a 30% increase in maximal strength for the triceps press and a 21% improvement for the horizontal rowing pull after 12 weeks. The improvements after a training period of 16 weeks in the study of Nash and colleagues [27] were 44% and 60% for similar exercises. A further investigation [28] found an increase of 45% for the vertical rowing pull after an intervention of only 6 weeks. However, the resistance used in this study corresponded to 75% of the 1RM, compared to the 50–60% of the 1RM in our previous study and the two other studies mentioned above. In general, several review articles have been published, which have shown that individuals with SCIs can achieve significant strength gains after an isolated strength-training intervention or experience benefits in strength as well as in general physical fitness if strength and endurance training are combined [7,9,13,18,20,23,24,29,30]. However, two reviews have shown [9,18] that most training studies are performed in individuals with chronic SCI and concluded that data for people with acute SCI (<1 year) are scarce, which makes a direct comparison of our data with most other studies difficult. However, in an older study [12], a group of eight individuals with chronic and five individuals with acute paraplegia were investigated. They performed different strength-training exercises three times a week for 16 weeks and the group showed significant increases between 10–46%. Unfortunately, there was no differentiation between the groups with chronic and acute SCI. Although this limits a direct comparison with our data and warrants further studies in people with acute SCI, it seems noteworthy to state that strength training is safe and feasible [18] and should and could be started even during primary rehabilitation. In this context, a clearly defined, supervised and standardized individual strength-training program can lead to significant improvements in maximal strength—a fact which was confirmed by our findings.

The present study also demonstrated some typical difficulties and challenges of training interventions in daily clinical practice. Five of the 17 participants enrolled at the beginning of the study could not be included in the final data analysis. The drop out reasons were urgent surgeries, cardiorespiratory complications, shoulder pain and very low training compliance, whereas 50% of the exclusions were related to shoulder problems. The high prevalence of shoulder complications reflects a well-known problem in the rehabilitation of people with SCI [31,32,33] and has to be taken into account by physicians and therapists. Thus, before the start of a specific strength-training program with weights, it has to be ensured that people are able to properly perform the exercises. Subsequently, a continuous, systematic and progressive training structure—ideally under the supervision of an experienced therapist—should be implemented. The documentation of each training session, as well as adequate training adaptations based on the periodical determination of the current training status (e.g., by means of the 1RM), are recommended as well. Such a strategy may help to find the balance between the strived-for constant performance enhancement and the risk of overuse injuries. This latter factor was the reason for determining the 1RM indirectly in this study, as according to Brzycki [25]. This procedure is most interesting if participants are not able to move the maximal weight for one single repetition due to health-related reasons or limited muscle coordination—a typical situation for people with SCIs during primary rehabilitation. It has been shown that based on the indirect determination of the 1RM, reliable information concerning maximal strength in individuals with SCIs can be obtained [34,35].

A further drawback of clinical training studies is training compliance. The present study found a very high compliance of 95%, which was clearly above the critical compliance rate of 80% reported in the literature [36,37]. Only one participant had to be excluded due to a training compliance of less than 50%. The reasons for the high compliance found in the present study can only be speculated; one reason might be that each patient was always accompanied by the same therapist, who was responsible for the determination of the 1RM, the training supervision and adaptations. This stands in contrast to situations with often-rotating therapists and seems to facilitate communication between the patient and the therapist. Possibly, signing a written informed consent form and acting as a study participant might have led to an additional boost in training motivation.

The main focus of this study was to determine the effects of a standardized strength-training program on the development of maximal strength in people with SCIs during primary rehabilitation. Besides this, the described strength-training intervention individuals also participated in their daily rehabilitation routine, which of course includes different, further rehabilitation activities; it is not possible to observe the isolated effects of a strength-training concept in such a setting. Although it seems reasonable to assume that the gains in maximal strength in our and other studies [12,38] were mainly related to the strength-training intervention, a certain contribution of other activities (e.g., arm cranking exercises) or spontaneous neurological recovery [39] cannot be fully excluded. However, this represents the normal daily clinical routine in a rehabilitation setting and at least underlines the value of the applied training programs in enhancing physical fitness in people with SCIs. In addition, the chosen study cohort included a heterogenous population of individuals with paraplegia with concern to, e.g., the completeness and level of the lesion (Table 1), which might have led to some variation concerning the outcome parameters. This method of recruitment was a conscious decision as it represents daily clinical practice at its best while also concomitantly underlining that a general strength-training concept might be suitable for different functional levels.

Nevertheless, in order to gain more detailed knowledge on strength-training adaptations, it seems desirable to investigate higher numbers and more homogenous patient groups with acute SCIs (e.g., including individuals with tetraplegia) during early rehabilitation in the future, as well as to elucidate what impact a gain in maximal strength will have on functioning, mobility, activities of daily life and well-being for these individuals. Finally, the present study only tested the impact of strength training on gains concerning the maximal strength of the muscles and muscle groups involved when using a triceps press and a horizontal rowing pull machine. Further projects might investigate if similar benefits might be reached with other exercises using different muscles and muscle groups or other training modalities (e.g., eccentric strength training).

Finally, the present work gives the first set of data on the level of the performance development of patients with acute paraplegia—an essential base for the calculation of statistical power for future, well-conducted clinical trials with this population.

## 5. Conclusions

A supervised and standardized strength-training program during the primary rehabilitation of people with paraplegia is feasible and leads to significant increases in maximal strength. Although study participants showed a high training compliance, factors such as medical complications may impede a proper implementation of a strength training concept into daily clinical practice.

## Figures and Tables

**Table 1:** Characteristics of study participants included at the start of the study (*n* = 17).

Patient	Gender	Age [y]	Height [cm]	Body Mass [kg]	Lesion	Lesion Level	AIS
1	f	29	176	68	traumatic	T8	B
2	m	61	168	69	traumatic	T12	A
3	m	68	172	82	non-traumatic	L2	D
4	f	42	163	82	traumatic	T6	B
5	f	48	179	60	non-traumatic	L1	B
6	m	24	178	52	traumatic	T6	A
7	f	67	167	54	traumatic	L1	C
8	m	62	170	59	traumatic	T4	B
9	m	43	185	85	traumatic	T12	A
10	f	83	159	93	non-traumatic	T9	D
11	m	57	180	75	traumatic	T3	A
12	m	20	173	70	traumatic	T1	D
13	f	55	168	55	traumatic	T10	A
14	m	26	175	51	traumatic	T12	D
15	m	47	165	76	traumatic	L2	B
16	m	56	180	81	traumatic	T6	B
17	m	62	177	68	traumatic	T3	A

AIS: American Spinal Injury Association Impairment Score; T: thoracic; L: lumbal; m: male. f: female.

**Table 2:** Development of the one repetition maximum (1RM) for the triceps and the horizontal rowing pull over the whole strength-training period.

	Triceps Pull	Horizontal Rowing Pull
1RM at		
T1 [kg] (Range)	45.3 (17.5–128.4)	47.7 (25.1–98.1)
T2 [kg] (Range)	52.3 (34.6–149.5)	57.0 (34.8–99.9)
T3 [kg] (Range)	67.9 (44.3–154.6)	64.3 (40.3–132.5)
*p*-value	0.003	<0.001
Effect size	0.48	0.82

1RM: one repetition maximum; T1-T3: Time point of measurements at baseline (T1), after 5–6 (T2) and 10–12 training weeks (T3).

## Data Availability

The datasets used and analyzed during the current study are available from the corresponding author on reasonable request.
